# Bioinformatics Analysis for Circulating Cell-Free DNA in Cancer

**DOI:** 10.3390/cancers11060805

**Published:** 2019-06-11

**Authors:** Chiang-Ching Huang, Meijun Du, Liang Wang

**Affiliations:** 1 Zilber School of Public Health, University of Wisconsin, Milwaukee, WI 53205, USA; 2 Department of Pathology and MCW Cancer Center, Medical College of Wisconsin, Milwaukee, WI 53226, USA; mdu@mcw.edu

**Keywords:** bioinformatics, copy number variation, cell-free DNA, methylation, mutation, next generation sequencing

## Abstract

Molecular analysis of cell-free DNA (cfDNA) that circulates in plasma and other body fluids represents a “liquid biopsy” approach for non-invasive cancer screening or monitoring. The rapid development of sequencing technologies has made cfDNA a promising source to study cancer development and progression. Specific genetic and epigenetic alterations have been found in plasma, serum, and urine cfDNA and could potentially be used as diagnostic or prognostic biomarkers in various cancer types. In this review, we will discuss the molecular characteristics of cancer cfDNA and major bioinformatics approaches involved in the analysis of cfDNA sequencing data for detecting genetic mutation, copy number alteration, methylation change, and nucleosome positioning variation. We highlight specific challenges in sensitivity to detect genetic aberrations and robustness of statistical analysis. Finally, we provide perspectives regarding the standard and continuing development of bioinformatics analysis to move this promising screening tool into clinical practice.

## 1. Introduction

To date, tissue biopsy samples are widely used to characterize tumors. Although tissues allow the histological definition of the disease and can reveal details of the genetic profile of the tumor, enabling prediction of disease progression and response to therapies, the applications are limited on tissue availability, sampling frequency, and their genetic heterogeneity [1]. Therefore, attention is turning to liquid biopsies, which enable the analysis of tumor components, including circulating tumor cells (CTC) [2] and circulating tumor nucleic acids from various biological fluids, mostly blood but also other easily accessible fluids such as urine [3]. Compared to conventional tissue biopsy from a single tumor site, the main advantages of liquid biopsies include their non-invasive characteristics, multiple sampling capability, and comprehensive coverage to address issues of tumor heterogeneity [4,5].

Circulating cell-free DNA (cfDNA) is defined as extracellular DNA occurring in blood or other body fluids. It is usually released as small fragments (150–200 bp in length [6]) from normal or tumor cells by apoptosis and necrosis [7], or shed from viable cells [8]. Levels of cfDNA are higher in diseased than healthy individuals [9]. cfDNA can track the evolutionary dynamics and heterogeneity of tumors and detect the early emergence of therapy resistance, residual disease, and recurrence [10,11,12]. Therefore, analysis of cfDNA has been considered as a potential screening approach for tumor diagnosis and prognosis by detecting tumor-associated aberrations in peripheral blood [13,14].

**Figure 1:**
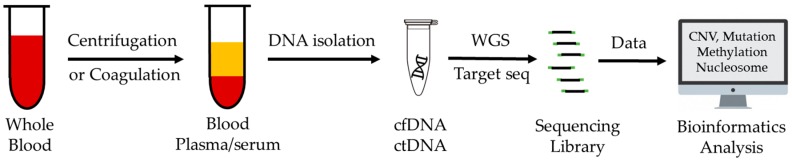
Workflow of blood-based liquid biopsy.

Next generation sequencing (NGS) has emerged as a powerful tool for cfDNA analysis, which allows the detection of cancer-related genetic and epigenetic alterations such as mutations, copy number variations (CNVs), and DNA methylation changes across wider genomic regions in many cancer types [15,16]. However, detection of cancer with high specificity and sensitivity is still challenging, especially in early-stage cancers, as there exist many barriers to the utilization of cfDNA in clinical applications, including lack of well-accepted sample collection protocol and sensitive detection approaches. Furthermore, analysis of cfDNA sequencing data requires specialized bioinformatics tools to identify robust biomarkers for clinical practice. In this review, we will discuss specific challenges in sensitivity to detect genetic aberrations and provide information on cfDNA bioinformatics approaches. We conclude with a perspective regarding future development in this rapidly evolving area. A simplified workflow of blood-based liquid biopsy is shown in Figure 1.

## 2. Characteristics of Circulating Tumor DNA (ctDNA)

The ctDNA is released from tumor cells only. The ctDNA can be derived from primary or metastatic tumors [17]. Most circulating ctDNA are 160–180 base pair fragments, roughly the size of a mononucleosomal unit [18,19]. However, recent studies have shown that ctDNA tends to be shorter than cfDNA from normal cells [20,21]. Therefore, ctDNA may be enriched by excising smaller DNA fragments from cfDNA on polyacrylamide gels [22]. Currently, cfDNA fragmentation patterns and their applications in liquid biopsy are an emerging research field. Although ctDNA can be used to detect the presence of cancer-related genetic and epigenetic changes, such changes usually vary from case to case, which makes the development of sensitive and generalizable approaches extremely challenging. One major challenge is low ctDNA fraction. In most cases, ctDNA accounts for a small fraction of total cfDNA since most cfDNA is derived from non-cancer cells, especially blood cells. In early-stage cancer patients, ctDNA fraction could be lower than 0.1%. To detect such a rare event with high specificity and sensitivity, a variety of approaches have been developed, which include droplet digital PCR (ddPCR) and molecular index-based next generation sequencing technologies [23,24].

## 3. Detection and Analysis of Somatic Mutations

Somatic mutations are involved in cancer development and progression. The presence or absence of a single genetic alteration in tumor DNA is currently employed to guide clinical decision making for a number of targeted agents [25,26,27,28]. Ever-increasing numbers of genomic alterations are being tested as putative predictive biomarkers in clinical trials of novel anticancer therapies [29]. To detect the cancer-associated alleles in the blood, real-time PCR (RT-PCR) and ddPCR “targeted” methods have been extensively adopted in most clinical trials [30]. Till now, clinical utility has been demonstrated for two FDA-approved cfDNA-based tests: the cobas epidermal growth factor receptor (EGFR) mutation test V2 (Roche Molecular Diagnostics), which detects EGFR mutation in plasma cfDNA from patients with lung cancer [31,32], and Epi proColon (Epigenomics AG), which reports on the methylation status of the Septin 9 promoter in plasma cfDNA from patients undergoing screening for colorectal cancer [33]. ddPCR is particularly useful to sensitively detect well-characterized mutations. The system can partition cfDNA into 20,000 nanoliter-sized droplets, where PCR amplification is carried out simultaneously. It is reported that the sensitivity of ddPCR can reach a limit of detection of 0.0005% BRAF V600E and V600K [34]. Another study reported that ddPCR can reliably detect AR-V7 expression from one spiked cell into 4000 lymphocytes (0.025%) [35]. Compared to the traditional NGS method, ddPCR is easier to use, has lower cost, and provides higher sensitivity and specificity. Although molecular barcoding technology has significantly increased the sensitivity and specificity of NGS, the low cost and easy-to-use features will make ddPCR widely accepted in clinical practice.

Although PCR-based assays can detect known mutations, the assay requires previous knowledge of target genes. In addition, the assay does not cover whole spectrum mutations in specific genes. Restriction of multiplexing capacity limits the simultaneous analysis of a large number of gene targets. Therefore, it may fail to identify less common but clinically relevant mutations. On the other hand, NGS, based on massive parallel sequencing of millions of different DNA molecules, allows the detection of multiple mutations in multiple genes. By using focused gene panels on clinically relevant targets, each nucleotide of interest can be sequenced thousands of times, ensuring a high degree of sensitivity. However, the requirement for such a high degree of sensitivity can easily lead to false positive results due to potential errors of PCR amplification and sequencing. To address this challenge, new data analysis approaches have been developed, among which is a new unique molecular identifier (UMI) strategy [36]. Another challenge related to mutation detection in cfDNA is to differentiate tumor mutations from background somatic mutations. Somatic mutations are common in healthy individuals with a rate between 2–6 mutations per 1 Mb [37]. Given the fact that the majority of cfDNA is from blood cells and ctDNA fraction in cancer patients is generally low, it is likely that most of the mutations identified in cfDNA could be irrelevant to cancer development, thereby impeding their clinical application [38,39,40]. This challenge points to the need for a large experiment to systematically investigate the mutation spectrum from both cfDNA and white blood cells in healthy and cancer patients.

## 4. Unique Molecular Identifier (UMI)-Based Target Sequencing

Target enrichment is a critical component of targeted deep sequencing for cost-effective, accurate, and sensitive detection of mutations, CNVs, and methylations in cfDNA. Common bioinformatics workflows allow sensitive and specific variant identification down to 2–5% allele frequency. This provides a sound methodology for identifying somatic mutations from solid tumor biopsies [41]. However, low ctDNA content in the blood and sequencing artifacts currently limit analytical sensitivity. In analyzing cfDNA from healthy controls, background errors are increasingly evident below allele fractions of ~0.2%. It is reported that under an allele fraction of 0.02%, >50% of sequenced genomic positions had artifacts [42]. In addition, common NGS assays involve multiple steps, including end repair, ligation, PCR, and sequencing. These steps often introduce technical biases, limiting accurate quantification and, therefore, hindering the robust and clinically valid detection of biomarkers [43]. Furthermore, PCR-based target enrichment cannot distinguish PCR duplicates from copies of unique fragments generated by a pair of PCR primers.

**Figure 2:**
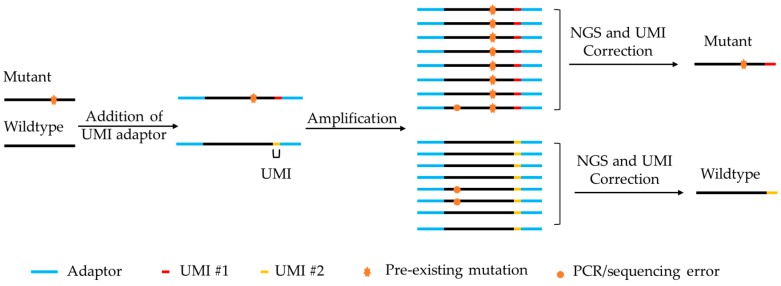
Principle of unique molecular identifiers (UMI) application in the detection of somatic mutations.

To overcome these limitations, UMIs (also known as molecular barcodes) have been added into the adaptors to tag individual DNA molecules [44,45,46,47]. Such barcodes enable the precise tracking of individual molecules. UMIs can accurately distinguish PCR duplicates from copies of unique fragments generated by PCR amplification [36]. Moreover, UMIs can reduce quantitative bias during experimental processes to detect true ultra-rare variants by distinguishing authentic somatic mutations arising in vivo from artifacts introduced ex vivo. This is largely due to the fact that errors arising from artifacts during library construction and sequencing runs could be eliminated by comparing the sequences of PCR duplicates identified with a UMI sequence [42,48]. Figure 2 illustrates the basic principle of UMI application in the detection of true somatic mutations. Dedicated bioinformatics software packages (Table 1) have been developed for the UMI-tagged targeted resequencing data to improve ultra-rare variant calling by removing errors arising from the first cycle PCR [49,50].

Incorporation of molecular barcoding into a bioinformatics algorithm has significantly increased sensitivity of mutation detection in NGS data. The detection sensitivity can be down to 0.01% [57]. However, recent advances in statistical modeling has also increased sensitivity of variant detection without molecular barcoding. A method ERAS-Seq (Elimination of Recurrent Artifacts and Stochastic Errors) that utilizes technical replicates in conjunction with background error modelling has shown an increased sensitivity of variant detection between 0.05% and 1% allele frequency [58]. By physically extracting and individually amplifying the DNA clones of erroneous reads, another barcoding-free method is reported to distinguish true variants of frequency >0.003% from the systematic NGS error. This method uses 10 times less sequencing reads compared to those from previous studies and achieved a PCR-induced error rate of 2.5 × 10^−6^ per base per doubling event [59].

## 5. Detection of DNA Copy Number Alterations

Currently, most cfDNA applications in cancer screening have focused on somatic point mutations [23,24]. However, methods that interrogate other genomic aberrations should be incorporated to improve detection and characterization of early-stage cancers. One of such genomic abnormalities is CNVs that contribute significantly to genome instability [60,61]. Large-scale cancer genome studies have identified CNVs across various types of cancer and a majority of the CNVs are shared among several cancer types [62,63]. Recently, several lines of investigation have demonstrated the potential of CNVs from cfDNA as sensitive cancer biomarkers [64,65,66]. Both targeted and whole genome sequencing (WGS) have been employed to identify specific CNVs or genome-wide DNA copy number patterns in cancer patients. Extension of statistical and bioinformatics methods developed from microarray-based comparative genomic hybridization (aCGH) array or NGS are suitable for the detection of CNVs from cfDNA.

For the WGS-based CNV analysis, depth of coverage (DOC) methods (Table 1) are the most used techniques to estimate copy number from the sequence depth in the genome [51,52,53,54]. Other methods such as assembly-based, split-read, and read-pair methods [67] can be used to infer copy number changes and chromosomal rearrangement. However, these methods may require high sequence coverage or specific molecular size and thus may not be practical in diagnostic application. The DOC methods can be divided into two major categories depending on whether a reference signal is required. In general, the pseudo-autosomal region on the Y chromosome and genomic regions with low mappability should be removed before the sequencing alignment procedure. This step is especially critical for reference free methods to ensure that the short reads can be mapped to a unique genomic location instead of multiple possible locations. The GEM (GEnome Multitool) mappability algorithm [68] is an efficient program that provides mappability information for multiple genomes. In addition, it is important to filter genomic regions that tend to show artificially high signal (i.e., excessive unstructured anomalous reads mapping). These blacklisted regions in the human genome are often found in highly variable regions (e.g., alternative haplotypes overrepresented on chromosome 19) or at specific types of problematic repeats such as centromeres, telomeres, and satellite repeats. The ENCODE and modENCODE consortia have identified these regions and made them available online [69] at https://sites.google.com/site/anshulkundaje/projects/blacklists. However, empirical data analysis indicates that the ENCODE blacklist may not be sufficient to remove all problematic regions. As such, the QDNAseq algorithm [51] provides a data-driven approach to identify additional regions that should be removed before downstream analysis.

Due to the high cost of WGS assay, current cfDNA-based approaches to CNVs detection normally have low-sequence coverage (e.g., 0.1×~0.5× coverage depth) [64,70,71]. As such, the binning procedure is generally required to aggregate reads mapped to a genomic window. After removing the low mappability reads and blacklisted regions, reads in different genomic windows are counted and normalized by the total number of reads. Depending on the read depth, a fixed bin size is normally chosen such that sufficient detection resolution can be achieved while excessive variation of read counts between adjacent windows can be reduced, thereby enhancing the detection sensitivity for CNVs. Although simple, using a fixed bin size may lead to high variability of read counts among bins with a substantially different number of mappable positions. To overcome this problem, the BIC-seq2 algorithm [53] normalizes read counts at a nucleotide level rather than at the bin level. It calculates the expected number of mapped reads for every position in the mappability map. The ratio of the observed read number and expected number of mappable reads is thus used to infer copy number for a specific genomic region. The normalized read counts can be further subject to GC content correction using smoothing techniques such as LOWESS [72]. The GC-corrected read counts are then normalized to the GC-corrected read counts of cfDNA from a group of reference samples (e.g., healthy controls or patient’s own germline DNA) and expressed as log_2_ ratio values. For reference-free methods, median normalization can be used to obtain log_2_ ratio values.

**Figure 3:**
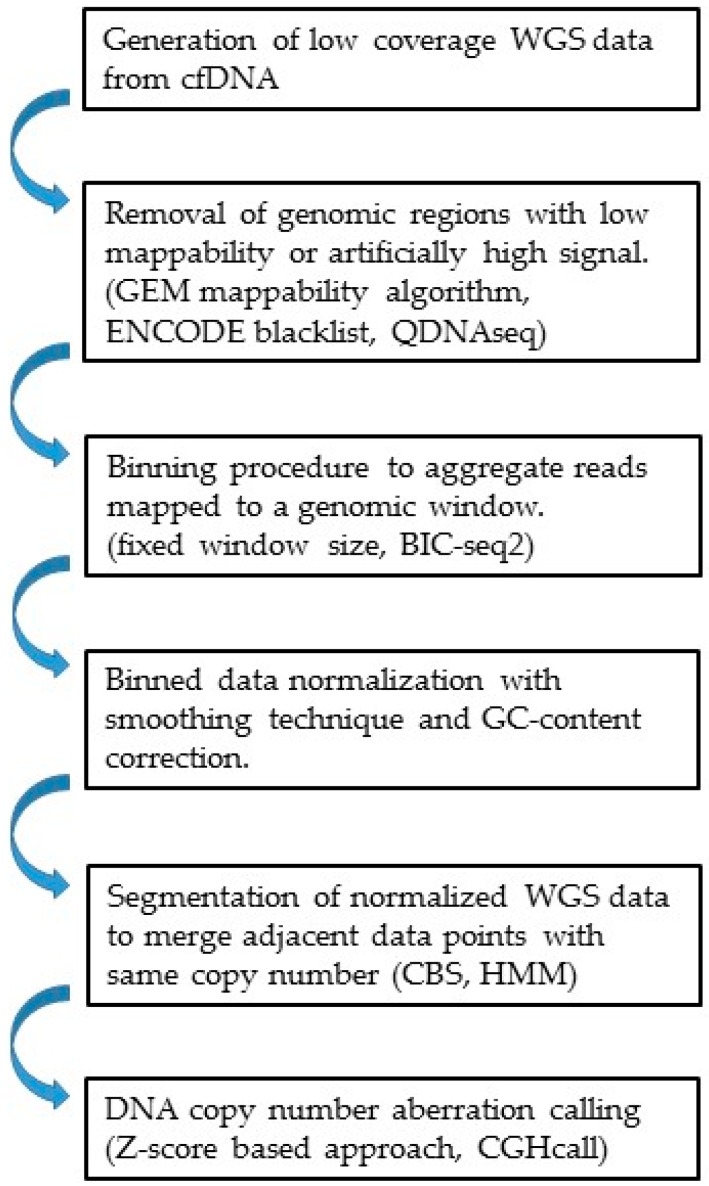
Bioinformatics procedure and techniques/resources used to detect copy number variations (CNVs) from low coverage whole genome sequencing (WGS) data.

Segmentation on the log_2_ ratio values is generally performed to identify the genomic areas with potential CNVs. The purpose of segmentation is to merge adjacent data points with the same copy number into one segment and divide regions with different copy numbers into different segments. Several statistical techniques and tools have been developed. Two of the most popular methods are circular binary segmentation (CBS) [73,74] and the hidden Markov model (HMM) [75,76]. Thorough review and systematic evaluation of CNV detection methods and software resources have been documented previously [52,77,78,79]. Researchers may use the information therein to choose appropriate algorithms for their projects. After the segmentation, aberration calling will be made to infer DNA regions with abnormal copy number (e.g., >2 or <2 DNA copies for gain or loss). A commonly used method for determining CNVs from the cfDNA of cancer patients using high throughput sequencing is the Z-score based approach [64,80,81,82]. These methods identify CNV segments by determining regions in the cfDNA that are significantly different from the reference panel (e.g., Z-score distribution from normal control). Other methods that make formal statistical inference for copy number are available [83,84]. For example, CGHcall [83] uses a two-level hierarchical mixture model to infer for each segment the likelihood of being one of six states of copy number: double deletion, single deletion, normal, gain, double gain, and amplification. This method uses log_2_ ratio data to estimate the proportion of different copy number states at the chromosome arm level. Therefore, it may require a large number of samples for robust inference, especially for chromosomes in which abnormal DNA copy numbers are rare. A summary of the bioinformatics procedure for WGS-based CNV analysis in cfDNA is shown in Figure 3.

One of the challenges to infer CNVs from the cfDNA sequencing data is attributable to the ctDNA content and tumor heterogeneity. In a large portion of cfDNA samples with low ctDNA content (i.e., <2%), especially in the early stages of cancer, sequencing reads are dominated by the DNA from non-cancer cells. Therefore, the signals of CNVs from cancer cells are almost entirely masked, leading to very little statistical power for any segmentation algorithms to detect CNVs, especially for focal amplifications or deletions. In addition, multiple clones of cancer cells could coexist in a cfDNA sample. This will make it even more difficult to detect CNVs due to genetic heterogeneity. To overcome this obstacle, Kirkizar et al. [85] developed a method that employs single-nucleotide polymorphism (SNP)-targeted massively multiplexed PCR (mmPCR) followed by NGS (mmPCR-NGS). Haplotype information is then obtained from the experiment to identify both single nucleotide variants (SNVs) and CNVs with high sensitivity and an average allelic imbalance as low as 0.5%. This method can also detect both clonal and subclonal CNVs in ctDNA.

## 6. Identification of DNA Methylation Changes from cfDNA

DNA methylation is essential for normal development and plays an important role in epigenetic control of gene activity. Changes in DNA methylation have been recognized as one of the most common molecular alterations in tumorigenesis [86,87]. It is well known that each tissue possesses unique methylation signatures and a genome-wide methylation pattern is distinguished between cancer and normal cells [16,88,89]. Therefore, whole genome methylation profiling from cfDNA could be a potentially powerful tool to detect the presence of specific cancer. Lehmann-Werman et al. [90] first demonstrated the feasibility to identify tissue origin using cfDNA. By leveraging whole genome methylation data sets from The Cancer Genome Atlas (TCGA) and Gene Expression Omnibus (GEO) repositories, they identified individual CpG dinucleotides that were unmethylated in the tissue of interest but methylated in other tissues. By comparing genome-wide methylation data from 35 human tissues generated using the Illumina Infinium HumanMethylation450k BeadChip, tissue-specific DNA methylation markers were selected. Subsequently, Moss et al. [91] generated a reference methylation atlas of 25 human tissues including major organs and cells involved in common diseases. For each tissue or cell type, both uniquely hypermethylated and uniquely hypomethylated CpG sites were identified. Additional CpG sites were further identified to differentiate any two cell types that were found to be most similar in the atlas.

With the data for tissue-specific and cancer methylation signatures, deconvolution algorithms [92], a commonly used algorithm to recover the original signal from a mixture of signal sources, can be used to map tumor tissue of origin from cfDNA. Sun et al. [93] used optimization programming to calculate the methylation densities of 5820 methylation markers in cfDNA from bisulfite sequencing data for 14 human tissues. To improve the selection of informative methylation markers, Guo et al. [94] identified 147,888 blocks of tightly coupled CpG sites, called methylation haplotype blocks, after a comprehensive analysis of a large amount of whole-genome bisulfite sequencing data, reduced-representation bisulfite sequencing data, and methylation array data. The deconvolution algorithm was then applied for tissue-specific methylation analysis at the block level. This method was successfully applied to estimate ctDNA content and differentiate among clinical plasma samples from normal individuals and patients of lung cancer and colorectal cancer.

Recently, probabilistic models have been formulated to identify specific cancer types from cfDNA. Kang et al. developed a method, termed CancerLocator [55], to simultaneously infer the proportion and tissue of origin of ctDNA using whole-genome DNA methylation data. By using TCGA Infinium HumanMethylation450 microarray data from both normal and tumor samples, CancerLocator identified as feature input a large number of CpG clusters that have high inter-individual methylation variation across all normal and cancer types. Since cfDNA from the peripheral blood is a mixture of normal and tumor DNA if a cancer cell is present, the methylation level for each CpG cluster, one for normal and the other one for a cancer type, can be estimated and the ctDNA fraction and the likelihood of the presence of a specific cancer type can be inferred based on the methylation data of informative CpG clusters. CancerLocator demonstrated a superior prediction performance over popular machine learning algorithms (i.e., random forest and support vector machine) on low-coverage sequencing data, especially for samples with low to moderate ctDNA fraction. However, a challenge facing this method is that the classification accuracy depends substantially on the estimated ctDNA fraction of a specific tumor type.

**Figure 4:**
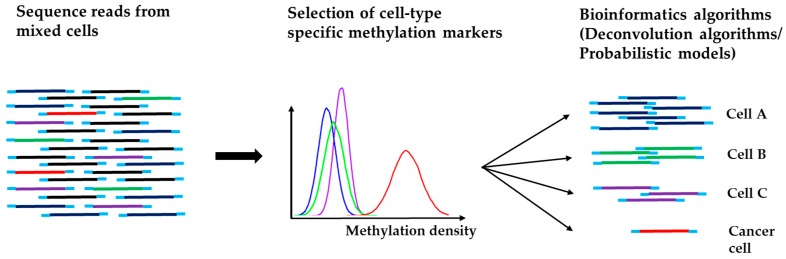
Schematic approach to map cancer tissue of origin from WGS methylation analysis.

A variation of CancerLocator was developed later by Li et al. [56]. This method, called CancerDetector, differs slightly from CancerLocator in genomic marker selection and estimation. To identify sensitive genomic markers, CpG clusters were identified such that the level of methylation in a specific cancer tissue differs from matched normal tissue as well as normal plasma samples. This procedure ensures that selected markers are not tissue specific and the methylation signal can be detected in the blood. With selected CpG clusters, a similar probabilistic model to CancerLocator was implemented to predict cancer types and ctDNA fraction. To improve the estimation of ctDNA fraction, an iteration procedure was developed to remove outlier markers whose estimated ctDNA fraction are far from the estimated ctDNA fraction when all markers were used. CancerDetector demonstrated substantial improvement over CancerLocator with high sensitivity and specificity in detecting tumor cfDNAs on real plasma data. Figure 4 illustrates the major principle of the bioinformatics approach for tumor tissue-specific methylation analysis.

## 7. Association of Nucleosome and Fragmentation Pattern with Tissue of Origin in cfDNA

In addition to DNA methylation, cfDNA fragmentation and/or nucleosome occupancy patterns are another epigenetic feature to trace gene activity and tissue origin [95]. Compaction of nucleosomal structures creates a barrier for DNA-binding transcription factors to access their cognate *cis*-regulatory elements. Usually, active promoters lack nucleosomes, while inactive promoters have densely packed nucleosomes. Nucleosome positioning through genome-wide mapping is shown to be associated with gene activation and expression in a development-dependent and tissue-specific manner [95,96]. Therefore, investigation of nucleosome positioning in a patient’s cfDNA may reveal the existence of a specific cancer type.

As cfDNA is preferentially released from apoptotic cells, the size distribution of cfDNA fragments (160–180 bp) can resemble the size of mononucleosome-protected DNA. Specifically, peak sizes correspond to nucleosomes (~147 bp) and chromatosomes (nucleosome + linker histone; ~167 bp), suggesting they could bear the information of the cell type of origin [97]. Based on the expectation that fragment endpoints should cluster next to nucleosome boundaries and should be depleted at sites of nucleosome occupancy, Snyder et al. showed that nucleosome spacing patterns can inform the cell type of origin from cfDNA [98]. The study showed that nucleosome spacing inferred from cfDNA in healthy individuals correlated strongly with epigenetic features of lymphoid and myeloid cells, consistent with hematopoietic cell death as a major source of cfDNA, while the patterns of nucleosome spacing in late-stage cancer patients match the anatomical origin of the patient’s cancer. Therefore, different nucleosome footprints between the tumor and the normal source of cfDNA may enable the noninvasive monitoring of a much broader set of clinical conditions than currently possible [98].

## 8. Conclusions and Future Direction

cfDNA molecules have emerged as promising biomarkers for cancer detection and monitoring due to the easy access to clinical samples from blood or urine. The advent of NGS technology provides an unprecedented opportunity to systematically examine the characteristics of cfDNA for tumor-specific changes. However, the massive amount of sequencing data requires sophisticated bioinformatics analysis to accurately identify genomic abnormalities in cancer. This review discussed major bioinformatics applications of cfDNA in oncological research to identify point mutations, copy number abnormalities, DNA methylation changes, and nucleosome positioning patterns. Using sophisticated bioinformatics analysis, advances have been made to better understand the property of cfDNA through fragmentation and nucleosome spacing patterns. Analysis by leveraging large-scale cancer genomic databases in conjunction with state-of-the-art statistical algorithms demonstrates the great potential of using methylation biomarkers for identification of cancer cell origin. Moreover, patterns of CNV through the WGS analysis can further reveal the extent of tumor heterogeneity. Nevertheless, to move cfDNA into routine clinical practices for better patient management, future studies will need to address several issues. First, studies need to focus more on detection sensitivity in early-stage cancer because there are many barriers to utilizing cfDNA for such applications. For example, most studies that demonstrated the feasibility of cfDNA in cancer detection used samples form late-stage cancer patients. However, the fraction of ctDNA in the plasma from early-stage cancer patients is generally very low. Although a range of NGS-based approaches have been used to characterize tumor genomes in detail and new bioinformatics techniques and analysis tools are rapidly evolving, current technologies and bioinformatics algorithms are not sensitive enough to detect such low level of genetic or epigenetic abnormalities. How to develop advanced technologies to detect mutations, CNVs, and epigenetic changes at the low ctDNA level is likely to be one of the most challenging issues to resolve. Another issue is related to cfDNA contaminations by the lysed blood cells and significant variation into cfDNA due to DNA isolation protocols and choice of instrument. Therefore, a standard protocol for quality control and bioinformatics analysis procedures need to be developed before these technologies can be successfully and reliably used in clinical practice and regulatory decision -making. A joint effort from the scientific community for the MicroArray Quality Control (MAQC) project [99] is an excellent example to follow to attain this goal. Finally, other biomarkers should be further explored for liquid biopsy in addition to genetic and epigenetic markers and nucleosome spacing patterns discussed in this review. For example, recent studies have shown that circulating cell-fee RNA (cfRNA), which encompasses miRNAs, lncRNAs, and mRNAs, could also serve as valuable biomarkers for liquid biopsy [100,101]. Given the finding that transcriptome profiling alone from tissue biopsies can robustly determine cancerous status and tissue origin [102], the multiparameter analyses incorporating the molecular profiles at cfDNA, cfRNA, and protein will result in an improved understanding of molecular aberrations and their functional roles across tumor types, as well as facilitate the identification of novel tumor subtypes [103]. As most of the cfDNA interrogations to date are proof-of-principle studies, large-scale, multi-site cohort studies that systematically investigate all these aspects of molecular profiles are needed to evaluate the complementary nature of their screening power so that liquid biopsy signatures can be refined, validated, and utilized in clinical practice. Eventually, these efforts will lead to the identification of new oncological biomarkers for early detection and outcome prediction, which is a prerequisite for realizing the promise of precision medicine.

## Figures and Tables

**Table 1:** Bioinformatics programs for detecting genetic and epigenetic changes in cancers.

Program	Website	Key Features	Reference
**Mutation**			
UMI-tools	https://GitHub.com/CGATOxford/UMI-tools	identifies sequencing errors in the UMI sequence to improve quantification accuracy	[49]
MAGERI	https://github.com/mikessh/mageri	provides an efficient analysis pipeline for UMI-encoded data	[50]
**Copy Number**			
QDNA-seq	https://github.com/ccagc/QDNAseq	simultaneously corrects for GC and mappability bias	[51]
WisecondorX	https://github.com/CenterForMedicalGeneticsGhent/WisecondorX	optimizes segmentation by reducing noise from problematic bins	[52]
BIC-seq2	http://compbio.med.harvard.edu/BIC-seq/	Avoids high variability of reads in bins	[53]
CNVkit	https://github.com/etal/cnvkit	uses both the targeted reads and the nonspecifically captured off-target reads to infer copy number	[54]
**Methylation**			
CancerLocator	https://github.com/jasminezhoulab/CancerLocator	simultaneously infers the proportion and tissue of origin of ctDNA	[55]
CancerDetector	https://zhoulab.dgsom.ucla.edu/pages/CancerDetector	Improves ctDNA fraction estimation and identifies outlier markers	[56]
